# A triclinic polymorph of (*E*)-2-(1-hy­droxy-3-phenyl­prop-2-en-1-yl­idene)-4,5-dimeth­oxy­cyclo­pent-4-ene-1,3-dione

**DOI:** 10.1107/S1600536812001043

**Published:** 2012-01-18

**Authors:** Masoumeh Hosseinzadeh, Mat Ropi Mukhtar, Mohammad Ali Khalilzadeh, Hamid Khaledi

**Affiliations:** a Centre for Natural Products and Drug Discovery, Department of Chemistry, Faculty of Science, University of Malaya, 50603 Kuala Lumpur, Malaysia; b Department of Chemistry, Science and Research Branch, Islamic Azad University, Mazandaran, Iran; c Department of Chemistry, University of Malaya, 50603 Kuala Lumpur, Malaysia

## Abstract

The title compound, C_16_H_14_O_5_, is a triclinic polymorph of a previously reported monoclinic structure [Hosseinzadeh *et al.* (2011 ▶). *Acta Cryst.* E**67**, o1544]. The mol­ecule is roughly planar, the r.m.s. deviation from the least-squares plane of all non-H atoms being 0.092 Å. In the crystal, adjacent mol­ecules are linked through C—H⋯O hydrogen bonds into an infinite two-dimensional network parallel to (011). The layers are further connected *via* C—H⋯π inter­actions, forming a three-dimensional structure. Intra­molecular O—H⋯O and C—H⋯O hydrogen bonds are also observed.

## Related literature

For the crystal structure of the monoclinic polymorph, see: Hosseinzadeh *et al.* (2011 ▶).
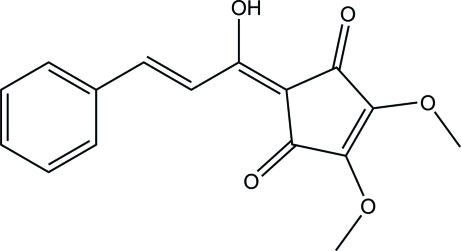


## Experimental

### 

#### Crystal data

C_16_H_14_O_5_
*M* *_r_* = 286.27
Triclinic, 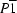

*a* = 5.4055 (2) Å
*b* = 11.2731 (3) Å
*c* = 11.6441 (3) Å
α = 72.070 (1)°
β = 83.088 (1)°
γ = 77.760 (1)°
*V* = 658.59 (3) Å^3^
*Z* = 2
Mo *K*α radiation
μ = 0.11 mm^−1^
*T* = 100 K
0.26 × 0.19 × 0.11 mm

#### Data collection

Bruker APEXII CCD diffractometer
Absorption correction: multi-scan (*SADABS*; Sheldrick, 1996 ▶) *T*_min_ = 0.973, *T*_max_ = 0.988
3340 measured reflections
2300 independent reflections
2041 reflections with *I* > 2σ(*I*)
*R*_int_ = 0.010

#### Refinement

*R*[*F* ^2^ > 2σ(*F* ^2^)] = 0.036
*wR*(*F* ^2^) = 0.098
*S* = 1.07
2300 reflections
195 parameters
H atoms treated by a mixture of independent and constrained refinement
Δρ_max_ = 0.18 e Å^−3^
Δρ_min_ = −0.25 e Å^−3^

### 

Data collection: *APEX2* (Bruker, 2007 ▶); cell refinement: *SAINT* (Bruker, 2007 ▶); data reduction: *SAINT*; program(s) used to solve structure: *SHELXS97* (Sheldrick, 2008 ▶); program(s) used to refine structure: *SHELXL97* (Sheldrick, 2008 ▶); molecular graphics: *X-SEED* (Barbour, 2001 ▶) and *XP* in *SHELXTL* (Sheldrick, 2008 ▶); software used to prepare material for publication: *SHELXL97* and *publCIF* (Westrip, 2010 ▶).

## Supplementary Material

Crystal structure: contains datablock(s) I, global. DOI: 10.1107/S1600536812001043/pv2504sup1.cif

Structure factors: contains datablock(s) I. DOI: 10.1107/S1600536812001043/pv2504Isup2.hkl

Supplementary material file. DOI: 10.1107/S1600536812001043/pv2504Isup3.cml

Additional supplementary materials:  crystallographic information; 3D view; checkCIF report

## Figures and Tables

**Table 1:** Hydrogen-bond geometry (Å, °) *Cg* is the centroid of C1–C6 ring.

*D*—H⋯*A*	*D*—H	H⋯*A*	*D*⋯*A*	*D*—H⋯*A*
O1—H1⋯O2	0.89 (2)	1.87 (2)	2.6802 (14)	150 (2)
C8—H8⋯O5	0.95	2.49	3.1015 (17)	122
C15—H15*B*⋯O3^i^	0.98	2.49	3.3751 (18)	151
C15—H15*C*⋯O2^ii^	0.98	2.49	3.3789 (18)	150
C16—H16*A*⋯O5^iii^	0.98	2.56	3.4143 (19)	145
C15—H15*A*⋯*Cg*^iv^	0.98	2.71	3.5728 (17)	147

Symmetry codes: (i) 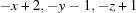; (ii) 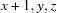; (iii) 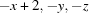; (iv) 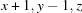.

**Fig. 1.**
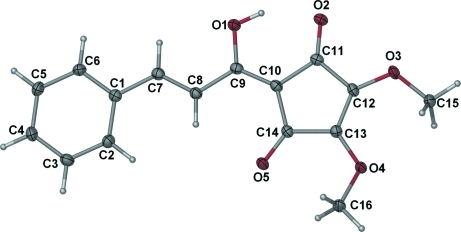
Molecular structure of the title compound with displacement ellipsoids drawn at 50% probability level. Hydrogen atoms are drawn as spheres of arbitrary radius.

**Fig. 2.**
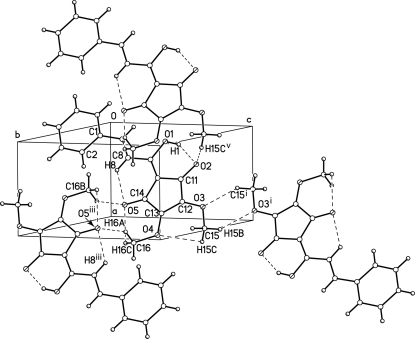
Intra- and intermolecular O—H···O and C—H···O hydrogen bonding in the structure. Symmetry codes: i = -x + 2, -y - 1; iii = -x + 2, -y, -z; v = x-1, y, z.

## supplementary crystallographic information

### Comment

The crystal structure of the title compound isolated from *Lindera pipericarpa* recrystallized from dichloromethane has been reported recently in monoclinic system with gross disorder (Hosseinzadeh *et al.*, 2011). In order to obtain a crystal of better quality, we recrystallized the compound from a different solvent, *i. e*., dimethyl sulfoxide (DMSO). The preliminary crystallographic data showed that the new crystals were formed in a triclinic system. The crystal structure of the new polymorph in the triclinic system is reported in this paper.

The title molecule (Fig. 1) is essentially planar [maximum atomic deviation = 0.2836 (13) Å for C16] and shows a higher deviation from planarity than is shown by the monoclinic structure [maximum atomic deviation = 0.064 (5) Å]. The crystal shows a three-dimensional supramolecular strucure formed by intermolecular C—H···O (Fig. 2) and C—H···π interactions (Table 1). In addition, intramolecular O—H···O and C—H···O hydrogen bonds are present.

### Experimental

The isolation of the title compound from *Lindera pipericarpa* (Lauraceae) has been reported recently (Hosseinzadeh *et al.*, 2011). Recrystallization of the title compound from DMSO at room temperature resulted in the formation of the triclinic polymorph.

### Refinement

The C-bound hydrogen atoms were placed at calculated positions and refined as riding atoms with H—C = 0.95 and 0.99 Å, for *sp*^2^ and methyl H-atoms, respectively. The O-bound H atom was located from a difference Fourier map and refined freely. For all H atoms *U*_iso_(H) were set to 1.2–1.5*U*_eq_(carrier atom).

### Crystal data

**Table:**

C_16_H_14_O_5_	*Z* = 2
*M**_r_* = 286.27	*F*(000) = 300
Triclinic, *P*1	*D*_x_ = 1.444 Mg m^−^^3^
Hall symbol: -P 1	Mo *K*α radiation, λ = 0.71073 Å
*a* = 5.4055 (2) Å	Cell parameters from 2073 reflections
*b* = 11.2731 (3) Å	θ = 2.3–29.6°
*c* = 11.6441 (3) Å	µ = 0.11 mm^−^^1^
α = 72.070 (1)°	*T* = 100 K
β = 83.088 (1)°	Block, orange
γ = 77.760 (1)°	0.26 × 0.19 × 0.11 mm
*V* = 658.59 (3) Å^3^	

### Data collection

**Table:**

Bruker APEXII CCD diffractometer	2300 independent reflections
Radiation source: fine-focus sealed tube	2041 reflections with *I* > 2σ(*I*)
graphite	*R*_int_ = 0.010
φ and ω scans	θ_max_ = 25.3°, θ_min_ = 2.3°
Absorption correction: multi-scan (*SADABS*; Sheldrick, 1996)	*h* = −6→4
*T*_min_ = 0.973, *T*_max_ = 0.988	*k* = −13→13
3340 measured reflections	*l* = −13→13

### Refinement

**Table:**

Refinement on *F*^2^	Primary atom site location: structure-invariant direct methods
Least-squares matrix: full	Secondary atom site location: difference Fourier map
*R*[*F*^2^ > 2σ(*F*^2^)] = 0.036	Hydrogen site location: inferred from neighbouring sites
*wR*(*F*^2^) = 0.098	H atoms treated by a mixture of independent and constrained refinement
*S* = 1.07	*w* = 1/[σ^2^(*F*_o_^2^) + (0.0544*P*)^2^ + 0.2122*P*] where *P* = (*F*_o_^2^ + 2*F*_c_^2^)/3
2300 reflections	(Δ/σ)_max_ < 0.001
195 parameters	Δρ_max_ = 0.18 e Å^−^^3^
0 restraints	Δρ_min_ = −0.25 e Å^−^^3^

### Special details

**Table:**

Geometry. All e.s.d.'s (except the e.s.d. in the dihedral angle between two l.s. planes) are estimated using the full covariance matrix. The cell e.s.d.'s are taken into account individually in the estimation of e.s.d.'s in distances, angles and torsion angles; correlations between e.s.d.'s in cell parameters are only used when they are defined by crystal symmetry. An approximate (isotropic) treatment of cell e.s.d.'s is used for estimating e.s.d.'s involving l.s. planes.
Refinement. Refinement of *F*^2^ against ALL reflections. The weighted *R*-factor *wR* and goodness of fit *S* are based on *F*^2^, conventional *R*-factors *R* are based on *F*, with *F* set to zero for negative *F*^2^. The threshold expression of *F*^2^ > σ(*F*^2^) is used only for calculating *R*-factors(gt) *etc*. and is not relevant to the choice of reflections for refinement. *R*-factors based on *F*^2^ are statistically about twice as large as those based on *F*, and *R*- factors based on ALL data will be even larger.

### Fractional atomic coordinates and isotropic or equivalent isotropic displacement parameters (Å^2^)

**Table:**

	*x*	*y*	*z*	*U*_iso_*/*U*_eq_	
O1	0.17619 (19)	0.04462 (10)	0.41452 (9)	0.0200 (3)	
H1	0.223 (3)	−0.0369 (18)	0.4540 (17)	0.030*	
O2	0.46326 (19)	−0.18778 (9)	0.47978 (9)	0.0197 (2)	
O3	0.9457 (2)	−0.32456 (9)	0.43272 (9)	0.0213 (3)	
O4	1.21886 (19)	−0.14869 (9)	0.22747 (9)	0.0204 (3)	
O5	0.84852 (19)	0.09819 (9)	0.16019 (9)	0.0203 (3)	
C1	0.0432 (3)	0.42536 (13)	0.20660 (13)	0.0175 (3)	
C2	0.2093 (3)	0.48875 (14)	0.11858 (13)	0.0204 (3)	
H2	0.3715	0.4439	0.1005	0.024*	
C3	0.1391 (3)	0.61581 (14)	0.05797 (13)	0.0217 (3)	
H3	0.2530	0.6575	−0.0018	0.026*	
C4	−0.0961 (3)	0.68287 (14)	0.08356 (14)	0.0231 (3)	
H4	−0.1438	0.7702	0.0412	0.028*	
C5	−0.2616 (3)	0.62210 (14)	0.17131 (15)	0.0251 (4)	
H5	−0.4224	0.6680	0.1897	0.030*	
C6	−0.1921 (3)	0.49427 (14)	0.23222 (14)	0.0215 (3)	
H6	−0.3065	0.4532	0.2922	0.026*	
C7	0.1065 (3)	0.29054 (13)	0.27304 (13)	0.0182 (3)	
H7	−0.0179	0.2566	0.3317	0.022*	
C8	0.3218 (3)	0.20994 (13)	0.25971 (12)	0.0173 (3)	
H8	0.4496	0.2399	0.2009	0.021*	
C9	0.3647 (3)	0.07895 (13)	0.33234 (12)	0.0165 (3)	
C10	0.5791 (3)	−0.00870 (13)	0.32366 (12)	0.0165 (3)	
C11	0.6157 (3)	−0.13911 (13)	0.40132 (12)	0.0163 (3)	
C12	0.8704 (3)	−0.20369 (13)	0.36824 (13)	0.0173 (3)	
C13	0.9804 (3)	−0.12165 (13)	0.27535 (12)	0.0165 (3)	
C14	0.8051 (3)	0.00484 (13)	0.24166 (12)	0.0159 (3)	
C15	1.1901 (3)	−0.39160 (13)	0.39974 (14)	0.0216 (3)	
H15A	1.1943	−0.3912	0.3152	0.032*	
H15B	1.2173	−0.4794	0.4520	0.032*	
H15C	1.3240	−0.3496	0.4097	0.032*	
C16	1.2894 (3)	−0.07311 (14)	0.10899 (13)	0.0235 (3)	
H16A	1.1761	−0.0760	0.0506	0.035*	
H16B	1.4646	−0.1066	0.0861	0.035*	
H16C	1.2757	0.0148	0.1094	0.035*	

### Atomic displacement parameters (Å^2^)

**Table:**

	*U*^11^	*U*^22^	*U*^33^	*U*^12^	*U*^13^	*U*^23^
O1	0.0194 (6)	0.0182 (5)	0.0188 (5)	−0.0031 (4)	0.0024 (4)	−0.0018 (4)
O2	0.0221 (6)	0.0194 (5)	0.0170 (5)	−0.0070 (4)	0.0012 (4)	−0.0029 (4)
O3	0.0243 (6)	0.0130 (5)	0.0212 (5)	−0.0004 (4)	0.0015 (4)	−0.0005 (4)
O4	0.0181 (6)	0.0179 (5)	0.0188 (5)	−0.0011 (4)	0.0028 (4)	0.0010 (4)
O5	0.0219 (6)	0.0161 (5)	0.0187 (5)	−0.0027 (4)	0.0019 (4)	−0.0007 (4)
C1	0.0187 (8)	0.0187 (7)	0.0162 (7)	−0.0039 (6)	−0.0020 (6)	−0.0059 (6)
C2	0.0188 (8)	0.0199 (7)	0.0209 (7)	−0.0007 (6)	0.0015 (6)	−0.0065 (6)
C3	0.0226 (8)	0.0202 (7)	0.0207 (8)	−0.0056 (6)	0.0028 (6)	−0.0041 (6)
C4	0.0235 (8)	0.0163 (7)	0.0258 (8)	−0.0011 (6)	−0.0030 (6)	−0.0020 (6)
C5	0.0177 (8)	0.0214 (8)	0.0323 (9)	0.0011 (6)	−0.0001 (6)	−0.0059 (7)
C6	0.0181 (8)	0.0205 (7)	0.0236 (8)	−0.0042 (6)	0.0023 (6)	−0.0040 (6)
C7	0.0195 (8)	0.0194 (7)	0.0162 (7)	−0.0051 (6)	−0.0010 (6)	−0.0048 (6)
C8	0.0183 (7)	0.0174 (7)	0.0155 (7)	−0.0039 (6)	−0.0009 (6)	−0.0035 (6)
C9	0.0188 (8)	0.0185 (7)	0.0135 (7)	−0.0059 (6)	−0.0001 (6)	−0.0049 (6)
C10	0.0202 (8)	0.0154 (7)	0.0139 (7)	−0.0045 (6)	−0.0010 (6)	−0.0034 (6)
C11	0.0201 (7)	0.0178 (7)	0.0133 (7)	−0.0063 (6)	−0.0010 (6)	−0.0058 (6)
C12	0.0220 (8)	0.0131 (7)	0.0163 (7)	−0.0030 (6)	−0.0028 (6)	−0.0032 (6)
C13	0.0175 (7)	0.0162 (7)	0.0157 (7)	−0.0023 (6)	−0.0012 (6)	−0.0052 (6)
C14	0.0184 (7)	0.0151 (7)	0.0146 (7)	−0.0037 (5)	−0.0026 (5)	−0.0042 (6)
C15	0.0201 (8)	0.0153 (7)	0.0256 (8)	−0.0011 (6)	0.0012 (6)	−0.0029 (6)
C16	0.0217 (8)	0.0226 (8)	0.0182 (8)	0.0001 (6)	0.0054 (6)	0.0005 (6)

### Geometric parameters (Å, °)

**Table:**

O1—C9	1.3447 (17)	C5—H5	0.9500
O1—H1	0.89 (2)	C6—H6	0.9500
O2—C11	1.2316 (17)	C7—C8	1.342 (2)
O3—C12	1.3396 (17)	C7—H7	0.9500
O3—C15	1.4480 (17)	C8—C9	1.443 (2)
O4—C13	1.3528 (17)	C8—H8	0.9500
O4—C16	1.4360 (17)	C9—C10	1.370 (2)
O5—C14	1.2244 (17)	C10—C11	1.4558 (19)
C1—C6	1.394 (2)	C10—C14	1.461 (2)
C1—C2	1.402 (2)	C11—C12	1.481 (2)
C1—C7	1.465 (2)	C12—C13	1.357 (2)
C2—C3	1.380 (2)	C13—C14	1.5026 (19)
C2—H2	0.9500	C15—H15A	0.9800
C3—C4	1.384 (2)	C15—H15B	0.9800
C3—H3	0.9500	C15—H15C	0.9800
C4—C5	1.387 (2)	C16—H16A	0.9800
C4—H4	0.9500	C16—H16B	0.9800
C5—C6	1.388 (2)	C16—H16C	0.9800
			
C9—O1—H1	107.7 (12)	C10—C9—C8	125.01 (13)
C12—O3—C15	118.30 (11)	C9—C10—C11	122.86 (13)
C13—O4—C16	119.37 (11)	C9—C10—C14	129.76 (13)
C6—C1—C2	118.33 (13)	C11—C10—C14	107.39 (12)
C6—C1—C7	118.69 (13)	O2—C11—C10	126.61 (13)
C2—C1—C7	122.98 (13)	O2—C11—C12	125.92 (13)
C3—C2—C1	120.58 (14)	C10—C11—C12	107.47 (12)
C3—C2—H2	119.7	O3—C12—C13	133.61 (13)
C1—C2—H2	119.7	O3—C12—C11	117.03 (12)
C2—C3—C4	120.50 (14)	C13—C12—C11	109.35 (12)
C2—C3—H3	119.8	O4—C13—C12	124.39 (13)
C4—C3—H3	119.8	O4—C13—C14	125.90 (12)
C3—C4—C5	119.72 (14)	C12—C13—C14	109.54 (12)
C3—C4—H4	120.1	O5—C14—C10	128.48 (13)
C5—C4—H4	120.1	O5—C14—C13	125.30 (13)
C4—C5—C6	119.99 (14)	C10—C14—C13	106.22 (11)
C4—C5—H5	120.0	O3—C15—H15A	109.5
C6—C5—H5	120.0	O3—C15—H15B	109.5
C5—C6—C1	120.88 (13)	H15A—C15—H15B	109.5
C5—C6—H6	119.6	O3—C15—H15C	109.5
C1—C6—H6	119.6	H15A—C15—H15C	109.5
C8—C7—C1	126.79 (13)	H15B—C15—H15C	109.5
C8—C7—H7	116.6	O4—C16—H16A	109.5
C1—C7—H7	116.6	O4—C16—H16B	109.5
C7—C8—C9	121.76 (13)	H16A—C16—H16B	109.5
C7—C8—H8	119.1	O4—C16—H16C	109.5
C9—C8—H8	119.1	H16A—C16—H16C	109.5
O1—C9—C10	119.60 (13)	H16B—C16—H16C	109.5
O1—C9—C8	115.38 (12)		

### Hydrogen-bond geometry (Å, °)

**Table:**

Cg is the centroid of C1–C6 ring.

**Table:**

*D*—H···*A*	*D*—H	H···*A*	*D*···*A*	*D*—H···*A*
O1—H1···O2	0.89 (2)	1.87 (2)	2.6802 (14)	150 (2)
C8—H8···O5	0.95	2.49	3.1015 (17)	122.
C16—H16C···O5	0.98	2.38	2.8600 (18)	110.
C15—H15B···O3^i^	0.98	2.49	3.3751 (18)	151.
C15—H15C···O2^ii^	0.98	2.49	3.3789 (18)	150.
C16—H16A···O5^iii^	0.98	2.56	3.4143 (19)	145.
C15—H15A···Cg^iv^	0.98	2.71	3.5728 (17)	147.

Symmetry codes: (i) −*x*+2, −*y*−1, −*z*+1; (ii) *x*+1, *y*, *z*; (iii) −*x*+2, −*y*, −*z*; (iv) *x*+1, *y*−1, *z*.
